# Combined treatment with basalt stone and placenta extract to improve the chronic pain and scar after breast cancer surgery: a case report

**DOI:** 10.1186/s13256-023-04264-7

**Published:** 2023-12-18

**Authors:** Mioka Inagaki, Eriko Otsuka, Yuko Hayashi, Masahiro Ohsawa, Eiichi Hirano

**Affiliations:** 1 WELLFIT, Co., Ltd., Tokyo, Japan; 2 Business Development Department, Japan Bio Products, Co., Ltd., Tokyo, Japan; 3 Department of Neuropharmacology, Graduate School of Pharmaceutical Sciences, Nagoya City University, Nagoya, Japan; 4 Medical Affairs Department, Japan Bio Products, Co., Ltd., 1-30-22 Maplewood Bldg., 3F, Tomigaya, Shibuya, Tokyo, 151-0063 Japan

**Keywords:** Surgical scars, Chronic pain, Basalt stone treatment, Placental extract, Case report

## Abstract

**Background:**

The changes in body image caused by breast deformities and postoperative pain have a detrimental influence on the physical and mental health of patients with breast cancer. The postoperative quality of life (QOL) of these patients reduces significantly owing to the changes in the breast, an organ unique to women, that occur following breast cancer surgery.

**Case presentation:**

This case report presents the case of a Asian woman in her early 40 s with postoperative hypertrophic scarring and contraction of the scar following mastectomy; the patient presented with decreased range of motion of the upper arm, hyperpigmentation from radiation burns, changes in breast shape, and chronic pain. The patient received a combination therapy comprising Basalt Stone Treatment and the application of horse placenta extract. As a result of a total of eight sessions conducted once every two weeks, the patient's pain and scar improved. No adverse events were observed after the therapy.

**Conclusion:**

Combination therapy with Basalt Stone Treatment and horse placenta extract improved the chronic pain and scar after breast cancer surgery.

## Background

The prevalence of breast cancer is increasing; however, the improvement in the treatment outcomes has led to patients seeking improved quality of life (QOL) after treatment [1, 2]. The changes in body image caused by surgical deformation of the breast and postoperative pain have a detrimental influence on the physical and mental health of patients with breast cancer. The postoperative QOL of these patients is decreased significantly owing to the surgical procedure performed on the breast, an organ unique to women [3]. In addition, the detrimental influence of breast cancer surgery on the activities of daily living, subjective perception of postoperative upper limb dysfunction, role performance, and psychological support have been reported to contribute to a decline in QOL [3–5]. Thus, it is essential to identify interventions that address the physical and mental aspects to improve the diminished QOL.

Sentinel node biopsy, axillary lymph node dissection [6], and radiation therapy to the axilla [7] may cause postoperative upper extremity dysfunction. Although most patients recover within the first postoperative year [8, 9], upper extremity dysfunction may persist for several years [10, 11]. Patients often require physically demanding treatments, such as radiotherapy and chemotherapy, postoperatively. A side effect of these treatments is impairment in mobility caused by lymphedema of the upper extremities, which limits day-to-day and social activities [6].

This case report describes a patient who underwent breast cancer surgery eight years ago and continued to suffer from chronic pain around the surgical scar for a long time. The patient received a treatment (hereafter referred to as "Basalt Stone Treatment") [12] that combines massage therapy using basalt (hereafter referred to as "basalt stone treatment") [13], which is developed based on the theory of Chinese Medicine Proof and lymphatic massage, and horse placenta extract [14], which is reported to have tissue regenerative effects. An improvement in physical and mental stress was observed owing to improved blood circulation. Repair of the surgical scar, which improved the stiffness around the scar, and the improvement in chronic pain and motor function.

## Case presentation

A Asian woman in her early 40 s was diagnosed with breast cancer in August 201X and underwent a partial right mastectomy in October 201X. A sentinel lymph node biopsy was performed to confirm the absence of metastasis. The combination with fluorouracil, epirubicin, and cyclophosphamide (FEC therapy) was initiated in December 201X. FEC therapy is a 21-day cycle of administering anticancer drugs. Once these 3 (three) anticancer drugs are administered on the first day, the remaining 20 days are a "rest period”. Thereafter, treatment proceeds in the same manner, in this case, continuing for four cycles. Specifically, fluorouracil was administered four doses of 150 mg each, epirubicin, and cyclophosphamide were administered for doses of 750 mg each. 43.2 Gy of Radiotherapy (16 times at 2.7 Gy) was initiated in March 201X + 1, followed by endocrine therapy with 11.25 mg of leuprorelin (once every 3 months), trastuzumab (once every 3 weeks; initial administration: 400 mg, after second time: 300 mg), and oral tamoxifen. 20 mg of Tamoxifen was administered until June 201X + 6. There was no recurrence of breast cancer. However, the patient experienced hardening, shrinkage, and deformation of the nipple; pain extending from the subclavian area to the right side of the neck; pain extending from the right side of the body to the site of the sentinel node biopsy; stiffness in the affected area; coldness; and darkening of the irradiated area. In addition, hyperpigmentation of the nipple due to the administration of anticancer drugs, induration of the right upper arm, and oedema on the right side of the body were also observed.

Recurrence of breast cancer was not observed in the 7 years that had passed since the surgery. However, the pain around the affected area persisted, which made it difficult for the patient to wear a seatbelt while driving. Unconscious clenching led to the occurrence of chronic headache on the right side of the head. Moreover, the range of motion of the right arm showed a significant reduction owing to oedema and subcutaneous tissue lumps caused by suture scar spasm and sentinel lymph node biopsy. Analgesic medications (60 mg of loxoprofen), stellate ganglion block injections administered at weekly intervals, and nerve root block injections (1% of Lidocaine) administered once a month when the pain was severe for six months provided transient pain relief, necessitating continuous pain management.

The patient underwent surgery in her early 40 s to promote surgical scar repair. The Basalt Stone Treatment comprised the following steps. Horse placenta extract (Japan Bio Products Co., Ltd., Tokyo, Japan) was splay-applied daily at two pushes per site on the sides of both breasts and around the suture scars, which were the areas that received radiation therapy. Basalt stone treatment was performed using the Cassa, Mushroom, Crescent, and Round stones. The Cassa Stone (Seven Beauty Co., Ltd., Tokyo, Japan) was used to lightly massage the back and pressure-sensitive chest area. The Mushroom Stone (Seven Beauty Co., Ltd., Tokyo, Japan) was used to lightly massage the back, intermammary sulcus, and neck, without the application of pressure, to relax the muscles. The Crescent Stone (Seven Beauty Co., Ltd., Tokyo, Japan) was used to lightly massage the muscles of the back, shoulder area, and neck to relax the muscles. The Round Stone (Seven Beauty Co., Ltd., Tokyo, Japan) was used to lightly massage the muscles, without the application of pressure, to relax the muscles and promote blood flow. Horse placenta extract (JBP) was applied at the end of the basalt stone treatment. The treatment was performed for a duration of approximately 110 min. The treatment site was managed according to the privacy policy stipulated by WELLFIT Co., Ltd.

In the present case, the placenta extract was re-applied after the treatment was completed. Basalt Stone Treatment was performed approximately once every two weeks for a total of eight times. The surgical scars were evaluated via visual observation and the measurement of the size (length and width) of the scars. Pain was evaluated by instructing the patient to select the best scale to indicate pain on the Visual Analogue Scale (VAS) [15]. A scale of 0 cm indicated pain-free state, whereas a scale of 10 cm indicated the strongest pain imaginable. The patient reported that the pain was not limited to the surgical scar, distributing to multiple sites in the surrounding area. Therefore, the pain score was evaluated as the sum of multiple sites where pain occurred. If the pain decreased in one area but increased in another area, the score was evaluated as the sum of all the additions and subtractions. The patient received a detailed explanation regarding the treatment procedure and measurements taken. Written consent was obtained before the procedure.

**Fig. 1:**
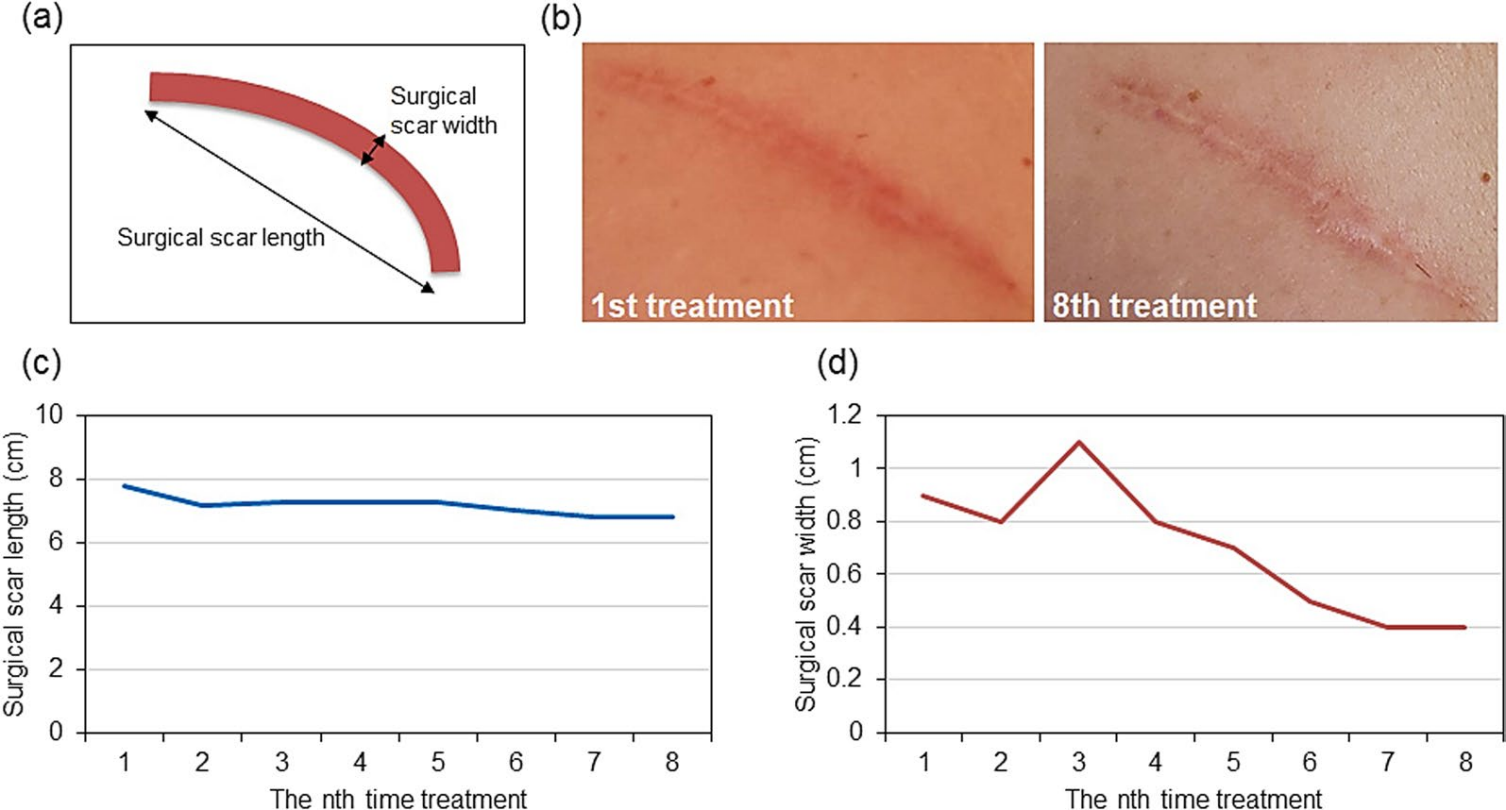
Changes in suture scar after basalt stone treatment and placenta extract application.** a** Schematic diagram of the length and width of the right subclavian surgical scar. **b** Suture scar area (left) at the first treatment session (day 1); suture scar area (right) at treatment 8 (day 127). **c** Changes in the length of the suture scar during the treatment period. **d** Changes in the width after removal of the suture scar during the treatment period. Suture length and width in cm

**Fig. 2:**
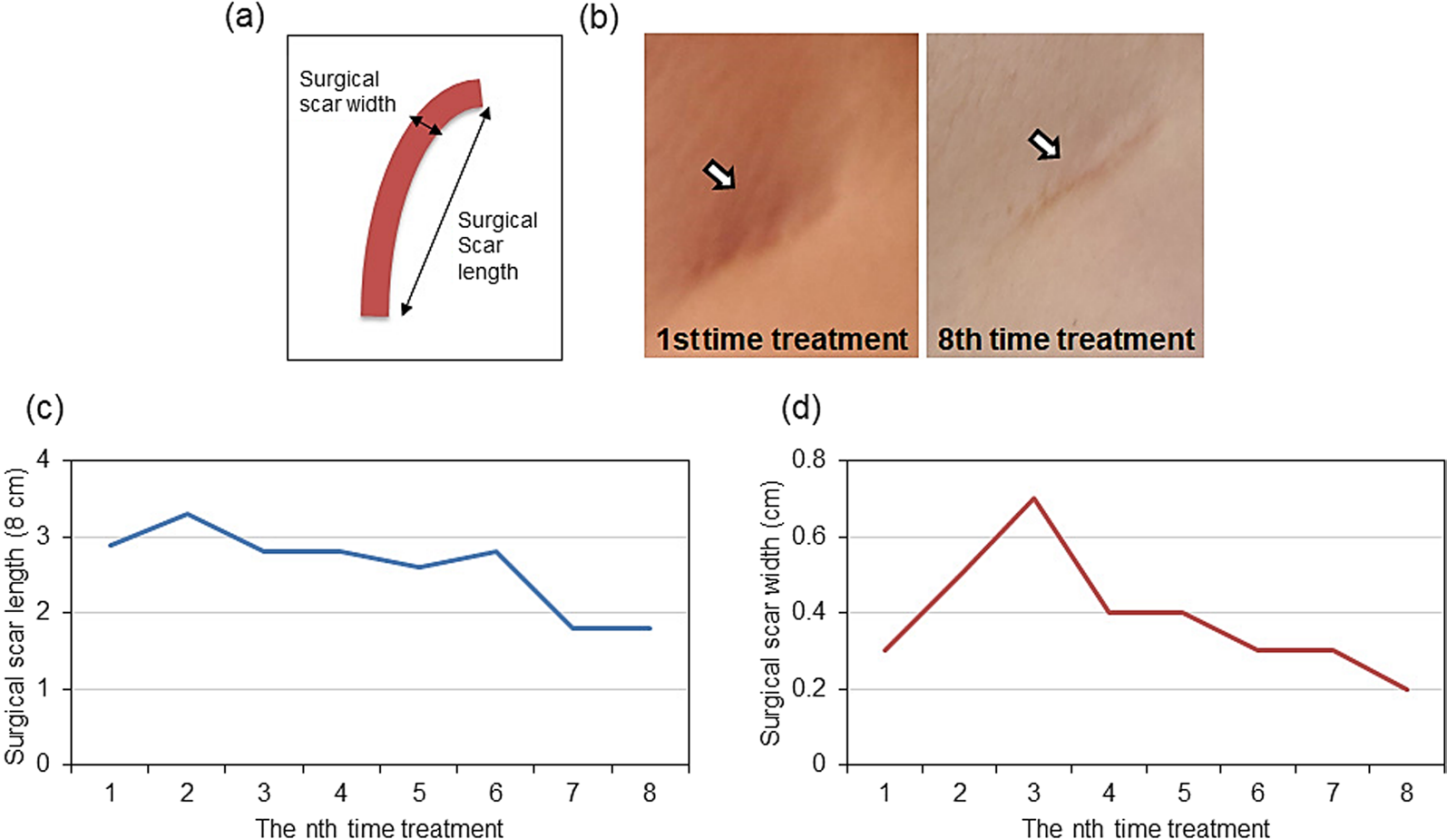
Changes in axillary suture scar caused by right axillary sentinel lymph node biopsy after basalt stone treatment and placenta extract application.** a** Schematic diagram of the length and width of the axillary surgical scar. **b** Axillary suture scar (left) at the first treatment session (day 1); axillary suture scar area (right) at the first eighth session (day 127).** c** Changes in the suture scar length during the treatment period. **d** Changes in the width of the suture scar during the treatment period. Suture length and width are expressed in cm

**Fig. 3:**
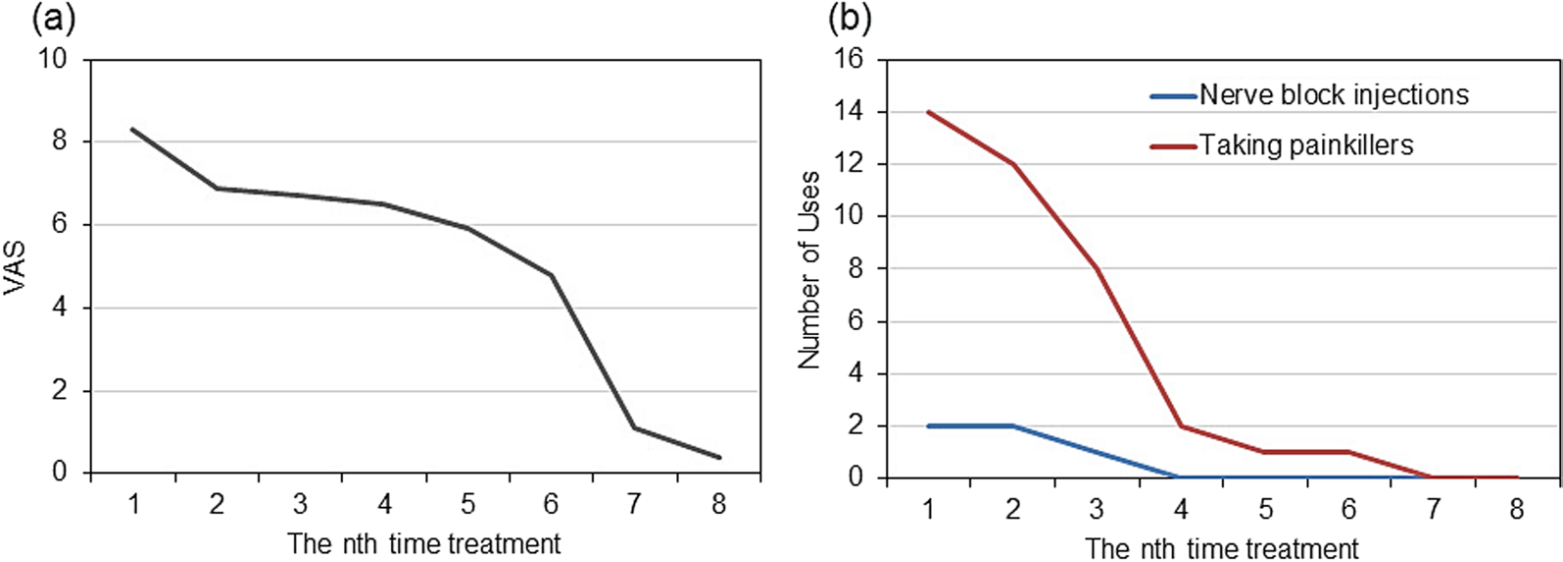
Changes in pain score after basalt stone treatment and placenta extract application. **a** Changes in the pain score from at the first treatment session (day 1) to the at the eighth treatment session (day 127). **b** Changes in the frequency of analgesic medication and block injection use from at the first treatment session (day 1) to the eighth treatment session (day 127)

The subacromial suture scar was erythematous at the first session (day 0), presumably due to inflammation at the site of the surgical scar and periphery of the scar (Fig. 1B, left). Erythema was observed at the site of the surgical scar at the eighth treatment session (127 days after the start of treatment); however, it had almost disappeared in the surrounding area (Fig. 1B, right panel). The subclavian suture scar was 7.8 cm long and 0.9 cm wide at the first session (day 0); in contrast, it was 6.8 cm long and 0.4 cm wide at the eighth session (day 127 from the start of treatment) (Fig. 1C, D, respectively). The axillary suture scar at the first session (day 0) showed a similar erythematous appearance, presumably due to inflammation at the site of the surgical scar and the surrounding areas (Fig. 2B, left panel). Erythema was observed at the site of the surgical scar at the eighth session (day 127 from the start of treatment); however, it had completely disappeared in the area surrounding the scar (Fig. 2B, right panel). The axillary suture scar was 2.9 cm long and 0.3 cm wide at the first session (day 0). In contrast, it was 1.8 cm long and 0.2 cm wide at the eighth session (day 127 from the start of treatment) (Fig. 2C, D, respectively). The pain score decreased from 8.3 to 6.7 (Fig. 3A) from the first to the third session (day 41 from the start of treatment), indicating that the cold sensation and pain in the subacromial surgical scar area and the stiffness of the subacromial surgical scar area was relieved. However, the surgical scar had to be covered and strong muscle tension on the right side of the back and hips and axillary stiffness were present. In addition, the pain in the surgical scar interfered with the daily life activities, requiring regular use of analgesics and weekly nerve block injections (Fig. 3B). Although the pain in the subclavian surgical scar was reduced, the pain at the site of the sentinel node biopsy, which had been the most painful site, increased. Similarly, the pain in the sternocleidomastoid muscle decreased; however, the pain in the chest and pectoralis major muscles, which were not noticeable earlier, increased. The pain score decreased from 6.7 to 4.8 from the third (41 days after the start of treatment) to the sixth session (83 days after the start of treatment) (Fig. 3A), and the patient was rarely disturbed by surgical scar pain. The axillary pain was relieved, and an improvement in the mobility of the area was also observed. The use of analgesics was reduced by 93% compared with the first treatment, and block injections were no longer necessary (Fig. 3B). However, an increase in pain in the latissimus dorsi, serratus anterior, and erector spinae muscles below the elbow was observed during inclement weather and loading on the operative side, particularly when carrying heavy loads. The frequency of clenching increased owing to pain, and pain expansion was observed in the temporal and sternocleidomastoid muscles. The pain score decreased from 4.8 to 0.4 from the sixth (83 days after the start of treatment) to the eighth session (127 days after the first treatment) (Fig. 3A). The disability in daily life due to pain at the surgical scar was resolved, and relaxation of muscle tension around the suture scar was observed. Furthermore, the tendency to roll the right shoulder and the habit of stretching movements that occurred during pain almost disappeared. No analgesic medications or block injections were administered (Fig. 3B). No significant adverse events were observed during the treatment.

## Discussion and conclusions

Surgical treatment of malignant tumours focuses on the removal of the tumour and treatment to control recurrence. Surgical treatment of malignant tumors centers on removal of the tumor and treatment to control recurrence. Breast cancer patients undergo breast surgery to remove the tumor. After breast cancer surgery, the scar site, chest, armpits, and inner arms may be painful and uncomfortable, as well as hot and swollen, even though several months or more have passed since the surgery. This condition is called Post-Mastectomy Pain Syndrome (PMPS) and is not uncommon and occurs in a certain percentage of people after breast cancer surgery [16–18]. The cause and pathogenesis of PMPS are not yet fully understood. It is speculated to be a type of neuropathic pain caused by injury to the intercostal nerves during surgery, while the surgical site may be hot and swollen for a long time, leading to chronic inflammation triggered by the surgery, which may cause ongoing pain. It has been suggested that gabapentin, non-steroidal anti-inflammatory drugs (NSAID), morphine, and their combinations are useful for initial pain control [19–21]. However, management of PMPS is often ineffective and there is no gold standard treatment guideline. Treatment of neuropathic pain includes a wide variety of medical, surgical, and alternative therapies. Identifying the most effective treatments available will help guide pain management in breast cancer patients and identify potential areas for further research and development. Since this patient had postoperative breast cancer pain for approximately 9 years, it can be inferred that the patient falls into the PMPS category. In addition, the patient did not respond to medical treatment, but showed improvement with Basalt Stone Treatment. Therefore, this treatment may be a new option for PMPS management. Although there are no indications on the efficacy of basalt stone treatment for pain, it has been reported that the administration of horse placenta extract to patients with osteoarthritis of the knee resulted in a significant reduction in pain and improvement in the swelling of the knee joint [22, 23]. Furthermore, placenta extract has been reported to have analgesic effects in patients with osteoarthritis of the knee, suggesting that these effects are mediated by cyclooxygenase-2 (COX-2) inhibition [23]. Thus, the significant improvement in the pain scores in the present case may be attributed to the analgesic effect of the placenta extract via COX-2 inhibition. However, it is possible that basalt stone treatment also contributed to the improvement as it has been reported that basalt stone treatment improves stiffness of the shoulders and back pain. However, the pain-relieving effects of basalt stones require further investigation.

Basalt Stone Treatment relieved tension on the right side of the body and improved the range of motion of the right arm. It has been reported that basalt stone treatment increases the blood flow velocity, sympathetic nerve arousal, and temperature of the body surface [12]. The relaxation of tension on the right side of the body and improvement in the range of motion of the right arm in the present case were attributed to Basalt Stone Treatment, as such effects have not been reported with the combination use of placenta extract. In addition, this treatment reduced the length and width of the suture scar and improved the colour tone of the suture scar and the burnt appearance of the irradiated area. Horse placenta extract has been reported to have wound healing and tissue regeneration effects [14]. Moreover, it promotes tissue turnover [16]. Thus, horse placenta extract may have contributed to these improvements. No single intervention that can comprehensively improve postoperative suture scars, muscle tension, associated decreased range of motion, and burnt appearance after radiotherapy, has been identified. The identification of such an intervention would improve the postoperative chronic pain such as PMPS.

For the use of placenta extract in the medical field, human placenta extract has been prescribed for about 50 years as a liver function-improving drug for chronic hepatitis, cirrhosis, and other liver diseases. Porcine placenta extract has also been reported to improve hot flashes, depression, fatigue, stiff shoulders, knee joint pain, and skin condition in perimenopausal and postmenopausal women [24–27], and is used in the gynecological field to relieve menopausal symptoms. In the present case, equine placenta extract was used. Although there is no report yet that horse placenta extract has shown the same clinical efficacy as human or porcine placenta extract, in the field of dermatology, it has been confirmed in human studies that horse placenta extract prevents UV-induced pigmentation [28]. Thus, medical use of horse placenta extract may be useful in the dermatological field.

In conclusion, the results of this case report indicate that combination treatment with basalt stone treatment and horse placenta extract may be an alternative cure for pain management in cases where postoperative pain is difficult to resolve with the administration of nerve blocks and analgesics is significantly impaired. In addition, this procedure may have notable benefits as an intervention with multifaceted effects that can be expected to promote postoperative suture scar repair and alleviate oedema. Although there are limitations to the simple comparison of the healing rate of suture scars and the superiority of events, such as muscle stiffness, oedema, and pain, using a control group, the usefulness of the procedure cannot be excluded. Nevertheless, further analysis of similar cases is required.

## Data Availability

All data generated or analysed during this study are included in this article.
